# Integrating Meteorological Data for Drought Prediction: A Hybrid Ensemble Machine Learning Approach in Northwestern Bangladesh

**DOI:** 10.1002/puh2.70440

**Published:** 2026-11-07

**Authors:** Sharmin Akther, Zafrin Sultana Shampa, Marufa Tasnim, Tasnia Ahsan Mohona, Mariam Tonni, Shajid Ahmed, Masuma Parvin, Md. Joni Alam

**Affiliations:** ^1^ Department of Software Engineering Daffodil International University Savar, Dhaka Bangladesh; ^2^ Department of Mathematics Comilla University Cumilla Bangladesh; ^3^ Department of Computer Science and Engineering Daffodil International University Savar, Dhaka Bangladesh; ^4^ Department of Statistics and Data Science Jahangirnagar University Savar, Dhaka Bangladesh

**Keywords:** Bangladesh, drought forecasting, hybrid ensemble model, Standardized Precipitation Evapotranspiration Index (SPEI), weighted average

## Abstract

**Background:**

Drought is a slow‐onset natural disaster with severe impacts on water resources, agriculture, and public health, particularly in climate‐vulnerable regions like northwestern Bangladesh. Accurate drought forecasting is essential for early warning and proactive mitigation.

**Methods:**

This study develops a hybrid ensemble machine learning framework for 7‐day‐ahead forecasting of the Standardized Precipitation Evapotranspiration Index (SPEI) in Dinajpur district, Bangladesh, using meteorological data from 2023 to 2025. Seven individual models—random forest (RF), Extreme Gradient Boosting (XGBoost), gradient boosting machine (GBM), support vector regression (SVR), long short‐term memory (LSTM), gated recurrent unit (GRU), and convolutional neural network (CNN)—were developed. Four hybrid configurations were constructed: simple average, weighted average, meta‐RF, and meta‐LR. An ablation study was conducted to evaluate the PM2.5 contribution.

**Results:**

Among individual models, GBM performed best (root mean square error [RMSE] = 0.0059, *R* ^2^ = 0.9999, Nash–Sutcliffe efficiency [NSE] = 0.9999), followed by RF (RMSE = 0.0079, *R* ^2^ = 0.9999, NSE = 0.9999) and XGBoost (RMSE = 0.0141, *R* ^2^ = 0.9997, NSE = 0.9996). These top three models (GBM, RF, and XGBoost) were selected as base models for hybrid ensembles. Among hybrid configurations, the Meta‐LR ensemble achieved the lowest RMSE (0.0050), whereas the weighted average ensemble (RMSE = 0.0052, *R* ^2^ = 0.9999, NSE = 1.0000) was selected as the final model due to its simplicity and statistically comparable performance. The ablation study revealed that PM2.5 did not consistently improve performance; therefore, it was excluded from the final model. The 2027 forecast predicts 75 drought days (20.5% of the year), with April identified as the most critical month (SPEI = −0.976).

**Conclusions:**

This study demonstrates that hybrid ensemble approaches provide accurate drought forecasts, with the weighted average model offering an optimal balance of performance and interpretability. The framework supports proactive public health interventions, water resource management, and climate‐resilient planning in drought‐prone regions.

## Graphical abstract

This study presents a hybrid weighted average ensemble model (GBM + RF + XGBoost) for 7‐day‐ahead SPEI drought forecasting in Dinajpur, Bangladesh. Among individual models, GBM performed best (RMSE = 0.0059, *R* ^2^ = 0.9999, NSE = 0.9999), followed by random forest (RMSE = 0.0079) and XGBoost (RMSE = 0.0141). The weighted average ensemble, combining these top three models with optimal weights (GBM = 0.546, RF = 0.408, XGB = 0.046), achieved superior performance (RMSE = 0.0052, *R* ^2^ = 0.9999, NSE = 1.0000), representing an 11.9% improvement over the best individual model. Although the meta‐LR ensemble achieved the lowest RMSE (0.0050), the weighted average was selected as the final model due to its simplicity and statistically comparable performance. An ablation study revealed that PM2.5 did not consistently improve model performance and was therefore excluded. The 2027 forecast predicts 75 drought days (20.5% of the year), with April identified as the most vulnerable month (SPEI = −0.976). The framework provides an early warning tool for public health interventions, including water quality monitoring, disease surveillance, and resource prepositioning.

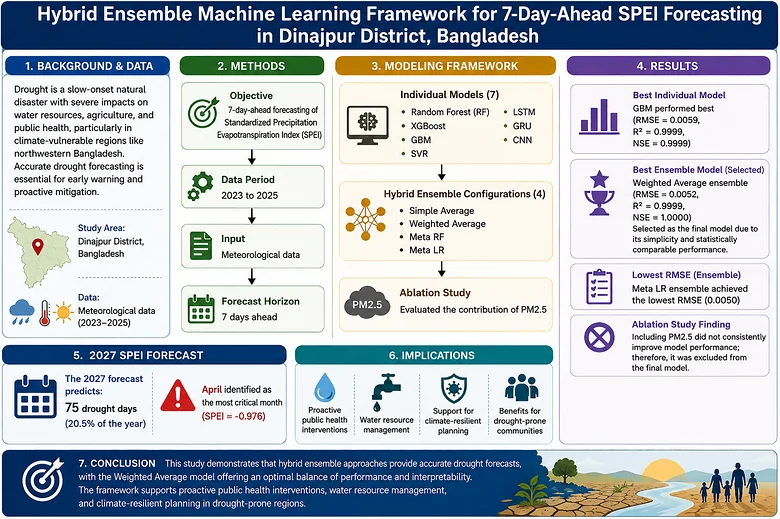

## 1 Introduction

Drought is a slow‐onset natural disaster with a profound impact on water resources, agricultural production, food security, and public health on a global scale [1, 2]. Unlike other hydrometeorological hazards such as floods and cyclones, drought develops gradually and persists over extended periods, making early detection and prediction particularly challenging [3]. Climate change, rising temperatures, and shifting precipitation patterns have intensified the frequency and severity of droughts worldwide, creating an urgent need for robust early warning systems to support drought mitigation and adaptation strategies [4, 5].

The People's Republic of Bangladesh is a country in South Asia, one of the most climate‐change‐susceptible countries in the world [6]. The country has significant monsoon rainfall, but there is a frequent occurrence of meteorological droughts, especially in the northwest part of the country [7, 8]. The region of Dinajpur district in the northwest of the country is worst hit by water scarcity during the pre‐monsoon season (March–May), which hurts rain‐fed agriculture, the recharging of groundwater resources, and the livelihoods of the rural population [9, 10]. Although Bangladesh is highly vulnerable to drought, the development of its drought forecasting is still in its infancy; most of the studies are on drought characterization, not prediction [11].

The Standardized Precipitation Evapotranspiration Index (SPEI) is a popular multi‐scale drought index that considers both precipitation and potential evapotranspiration and is sensitive to drought intensification due to the effects of climate change on temperature patterns [4]. The SPEI incorporates the atmospheric water demand, not just rainfall, to get a more complete picture of drought conditions in a warmer climate compared to only the Standardized Precipitation Index (SPI) [12]. But the nonlinear and stochastic nature of SPEI is a challenge for conventional statistical forecasting techniques [11].

Machine learning (ML) and deep learning (DL) methods have shown great promise for time series forecasting in hydrology and climatology [13, 14]. Models, such as random forest (RF), Extreme Gradient Boosting (XGBoost), gradient boosting machine (GBM), support vector regression (SVR), long short‐term memory (LSTM), gated recurrent unit (GRU), and convolutional neural network (CNN), have been successfully applied for drought forecasting [15, 16], whereas ensemble methods like weighted averaging and stacking have proven more effective by combining multiple models [17]. Ensemble forecasting has also been successfully applied to public health in Bangladesh, including weighted ensemble for COVID‐19 [18] and dengue transmission, identifying a 71‐day peak window with an estimated 11,500 cases for 2026 [19]. Recent studies across diverse climatic regions have further reinforced the effectiveness of ML‐based drought prediction. In Türkiye, wavelet‐transformed ML models achieved superior streamflow forecasting performance, with SVM after wavelet transformation yielding correlation coefficients up to 0.9846 and Nash–Sutcliffe efficiency (NSE) values of 0.9695 [20]. In Norway, wavelet‐enhanced LSTM models provided the best drought index forecasts across multiple regions, achieving correlation coefficients of 0.9765 and NSE values of 0.9510 [21]. Similarly, comparative evaluations of SVM, LSTM, and XGBoost for SPEI forecasting across Norwegian regions demonstrated that model performance varies significantly by location and input structure, with SVM achieving the highest NSE (0.8954) for certain regions [22]. Recent advances in hybrid and ensemble forecasting have improved predictive accuracy across environmental and public health applications. An evolutionary hybrid RF model for SPEI forecasting improved accuracy by 30%–40% over classic RF [23]. Similarly, an ensemble framework combining statistical and ML approaches for global monkeypox forecasting demonstrated that weighted and equal ensembles consistently outperformed individual models [24]. However, despite these advances, ensemble approaches have not been systematically applied to drought prediction in Bangladesh, nor has air quality data (PM2.5) been rigorously evaluated through ablation studies in this context.

This study addresses these gaps by developing and evaluating a hybrid weighted average ensemble model (WEBM) combining GBM, RF, and XGBoost for 7‐day‐ahead SPEI forecasting in Dinajpur, Bangladesh. Specifically, this research (i) calculates SPEI from daily meteorological data (2023–2025), (ii) evaluates the predictive contribution of PM2.5 through an ablation study, (iii) develops and tunes seven individual ML/DL models, (iv) constructs four hybrid ensemble configurations (simple average, weighted average, meta‐RF, and meta‐LR), and (v) forecasts SPEI for the full year 2027 to support drought early warning and public health preparedness.

## 2 Methods and Materials

### 2.1 Study Area and Data Source

This study was conducted for the Dinajpur district, located in the drought‐prone northwestern region of Bangladesh (25.63° N, 88.64° E) (Figure 1) [25]. The district covers approximately 3444 km^2^ and features a low‐lying alluvial plain with an average elevation of 35–40 m above sea level [26]. The region experiences a tropical monsoon climate with distinct seasonal variations [27]. The annual average temperature is 25.1°C, ranging from 16–18°C in winter (December–February) to 28–30°C in summer (April–September). The district receives approximately 1720 mm/year of rainfall, with 70%–75% occurring during the southwest monsoon (June–September). The major rivers draining the region include the Atrai, Punarbhaba, and Dhepa, which are part of the Ganges–Brahmaputra river basin system [27, 28]. This system governs the region's surface water hydrology, which, in turn, influences groundwater recharge, agricultural practices, and the overall water balance used in SPEI calculations. Figure 1 presents the spatial distribution of mean SPEI and mean PM2.5 across Dinajpur district during the study period (2023–2025). The SPEI map reveals a distinct spatial gradient, with values ranging from −0.65 in the southeastern region to +0.45 in the central‐northern region, indicating spatial heterogeneity in drought conditions. The overall mean SPEI of −0.0003 indicates near‐normal conditions over the study period. The PM2.5 map shows concentrations ranging from 35 to 65 µg/m^3^, with a district‐wide mean of 50.28 µg/m^3^, exceeding the WHO annual guideline of 25 µg/m^3^. Higher PM2.5 concentrations are observed in the southern and central parts of the district, whereas the northern regions exhibit relatively lower concentrations.

**FIGURE 1:**
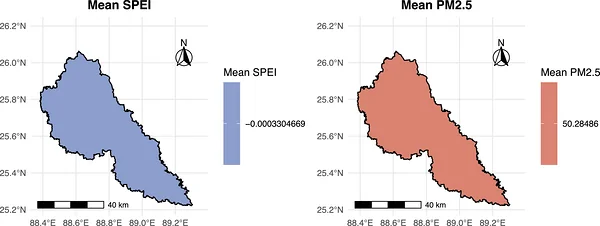
Spatial distribution of mean SPEI (left panel) and mean PM2.5 (right panel) across Dinajpur district, Bangladesh, during the study period (2023–2025). SPEI, Standardized Precipitation Evapotranspiration Index.

Daily meteorological and air quality data were obtained from a publicly available dataset covering all 64 districts of Bangladesh [29]. The dataset was sourced from the Open‐Meteo API, which provides historical weather and air quality reanalysis data. For this study, data for Dinajpur district were extracted, comprising 1096 daily observations from January 1, 2023, to December 31, 2025. The dataset included the following variables: daily average temperature (°C), total daily rainfall (mm), daily maximum wind speed (km/h), and daily average PM2.5 concentration (µg/m^3^). No missing values were present in the extracted dataset.

Figure S1 shows the monthly variations in (a) precipitation and (b) PM2.5 concentrations across Dinajpur district (2023–2025). The figure clearly illustrates the inverse relationship between rainfall and PM2.5, with monsoon months (June–September) showing peak precipitation and lowest PM2.5 concentrations (25–35 µg/m^3^), whereas winter months (December–February) exhibit minimal rainfall and maximum PM2.5 levels (70–80 µg/m^3^. The WHO guideline of 25 µg/m^3^ is indicated for reference. Table 1 summarizes the comprehensive dataset specifications, including variables, measurement methods, and quality control procedures.

**TABLE 1:** Comprehensive dataset specifications for Dinajpur district study.

Feature	Specification	Details
Time period	2023–2025	3‐year monitoring period
Temporal resolution	Daily	1096 daily observations
Spatial coverage	88.0–89.5° E, 25.0–26.5° N	Dinajpur district, Bangladesh
Data split	Walk‐forward validation	Training: 2023–2024 (688 days); testing: 2025 (365 days)
Target variable	SPEI	Standardized Precipitation Evapotranspiration Index
Precipitation	mm/day	Daily rainfall measurement
Temperature	°C	Daily average temperature
Wind speed	km/h	Daily maximum wind speed
Data source	Open‐Meteo API	Historical weather and air quality reanalysis data
Features	25 meteorological features	Rainfall, temperature, wind speed with lagged (1, 3, 7, 14, and 30 days) and rolling (7, 14, and 30 days) statistics; seasonal features (Month_sin, Month_cos, Day_sin, Day_cos)
Derived variables	SPEI	Calculated following Vicente‐Serrano et al. using the Thornthwaite method
Quality control	Outlier removal, gap filling	Linear interpolation (<5% missing data)
Data processing	Seasonal aggregation	Winter, pre‐monsoon, monsoon, post‐monsoon

### 2.2 SPEI Calculation

The SPEI was selected as the drought index because it accounts for both water supply (precipitation) and atmospheric water demand (potential evapotranspiration, PET), making it sensitive to temperature‐driven drought intensification under climate change [4].

SPEI was calculated following the standardized methodology of [4]. First, PET was estimated using the Thornthwaite method [30], which requires only monthly mean temperature and station latitude (25.63° N for Dinajpur). The monthly heat index (*i*) was computed as follows:

(1) $$i={\left(\frac{T}{5}\right)}^{1.514}$$

where *T* is the monthly mean temperature (°C). The annual heat index (*I*) is the sum of the 12 monthly values:

(2) $$I=\sum_{1}^{12}i$$

The annual heat indices were 137.53, 137.07, and 137.18 for 2023, 2024, and 2025, respectively. The PET for each month was then calculated as follows:

(3) $$\mathrm{PET}=16\times {\left(\frac{10T}{I}\right)}^{\alpha }\times \frac{N}{12}\times \frac{d}{30}$$

where *α* is a coefficient derived from *I*, *N* is the number of days in the month, and *d* is the mean daily daylight hours for the month at the station latitude. Monthly PET values were downscaled to daily PET by dividing by the number of days in each month.

A 30‐day rolling window was applied to compute the water balance (*D*):

(4) $$D=P_{30}\;-\mathrm{PE}T_{30}$$

where *P*_30_ is the 30‐day accumulated rainfall, and PET_30_ is the 30‐day accumulated PET. The *D* series was fitted to a log‐logistic probability distribution, and the cumulative distribution was transformed to a standard normal distribution to obtain SPEI values. The first 29 days were excluded from the final dataset due to the 30‐day rolling window requirement.

### 2.3 Feature Engineering

To develop the forecasting model, the target variable was defined as SPEI at *t* + 7 days. Lagged features (1, 3, 7, 14, and 30 days) were created for rainfall, PM2.5, and temperature to capture temporal dependencies. Additionally, rolling statistics (7, 14, and 30‐day windows) were computed for rainfall, PM2.5, and temperature. Seasonal features, including month and day‐of‐year, were encoded using sine and cosine transformations to capture cyclic patterns. A total of 32 features were initially constructed, including PM2.5‐related variables. On the basis of the ablation study results, PM2.5 was excluded from the final model, and the feature set was reduced to 25 meteorological features (rainfall, temperature, wind speed, and their lagged/rolling statistics) for the final forecasting framework. It is important to note that water balance (precipitation − PET) was included as a predictor variable. As SPEI is fundamentally a standardized transformation of water balance, this creates an inherent near‐deterministic relationship between the predictor and target variables. This explains the exceptionally high‐performance metrics (*R* ^2^ ≈ 1.0000, NSE ≈ 1.0000) observed for tree‐based ensemble models, as they essentially reconstruct the standardized drought index from its underlying components rather than forecasting from independent predictors.

### 2.4 Data Splitting and Normalization

The dataset was chronologically split using walk‐forward validation to ensure temporal consistency and avoid data leakage. Data from 2023 to 2024 (688 days) were used for training and hyperparameter tuning, whereas data from 2025 (365 days) were reserved for independent testing. This approach simulates a real‐world forecasting scenario where models are trained on historical data and evaluated on future unseen data, which is critical for operational drought prediction systems [31].

All features were normalized to the [0, 1] range using Min–Max scaling to ensure equal contribution from variables with different units and to improve neural network convergence [32]:

(5) $$\;X_{\mathrm{norm}}=\frac{X-X_{\min}}{X_{\max}-X_{\min}}\;$$

### 2.5 ML Models

Four ML models were developed for SPEI forecasting: RF, XGBoost (XGB), GBM, and SVR. The selection of these models was based on their proven success in hydrological and drought forecasting applications [33, 34, 35, 36, 37, 38]. A summary of their advantages, disadvantages, and suitability for hydrological applications is provided in Table 2.

**TABLE 2:** Comparison of machine learning models for hydrological applications, including their advantages, disadvantages, and hydrological suitability.

Model	Advantages	Disadvantages	Hydrological suitability
Random forest (RF)	Robust to overfitting; handles nonlinearity; provides feature importance; resistant to noise	Computationally expensive for large datasets; limited extrapolation	Excellent for capturing complex rainfall‐runoff and drought relationships
XGBoost	High efficiency; handles missing data; built‐in regularization; strong predictive accuracy	Requires careful hyperparameter tuning; sensitive to noisy data	Highly effective for drought prediction with large datasets; captures complex nonlinear interactions
Gradient boosting machine (GBM)	Flexible; high predictive accuracy; handles various data types	Prone to overfitting without regularization; computationally intensive	Suitable for capturing seasonal drought patterns and hydrological extremes
Support vector regression (SVR)	Effective in high‐dimensional spaces; kernel flexibility	Less interpretable; sensitive to parameter selection; weaker performance in some hydrological applications	Useful for small to medium datasets with nonlinear relationships

#### 2.5.1 Random Forest

RF is an ensemble learning method that constructs multiple decision trees during training and outputs the mean prediction of the individual trees [39]. For a regression problem with *n* trees, the RF prediction is given by the following equation:

(6) $$\;\hat{y}=\frac{1}{n}\;\sum_{i=1}^{n}T_{i}\left(x\right)$$

where $T_{i}\left(x\right)$ is the prediction of the *i*th decision tree. RF is robust to overfitting and captures complex nonlinear relationships between predictors and SPEI.

#### 2.5.2 XGBoost

XGBoost (Extreme Gradient Boosting) is an optimized distributed gradient boosting algorithm designed for efficiency and predictive performance [40]. It builds trees sequentially, where each new tree corrects errors made by previous trees. The prediction is given by the following equation:

(7) $$\;{\hat{y}}_{i}=\sum_{k=1}^{K}f_{k}\;\left(x_{i}\right),f_{k}\in F$$

where *K* is the number of trees and $f_{k}$ are the individual tree functions. XGBoost incorporates regularization to prevent overfitting [18].

#### 2.5.3 Gradient Boosting Machine

Gradient boosting builds an ensemble of weak prediction models, typically decision trees, in a stage‐wise fashion [41]. At each iteration *m*, a new tree is added to predict the residuals of the previous ensemble:

(8) $$F_{m}\left(x\right)=F_{m-1}\;\left(x\right)+\rho_{m}h_{m}\left(x\right)$$

where $h_{m}$ is the newly added tree, and $\rho_{m}$ is the learning rate.

#### 2.5.4 Support Vector Regression

SVR is a kernel‐based method that maps input features into a high‐dimensional space to find a function that deviates from the actual target values by a value no greater than *ε* [42]. The SVR prediction function is as follows:

(9) $$f\left(x\right)=\sum_{i=1}^{n}\left(\;\alpha_{i}-\alpha_{i}^{*}\right)K\left(x_{i},x\right)+b$$

where $\alpha_{i}$ and $\alpha_{i}^{*}$ are Lagrange multipliers, $K(x_{i},x)$ is the kernel function (radial basis function [RBF] used here), and *b* is the bias term.

### 2.6 DL Models

Three recurrent neural network (RNN) architectures were developed: LSTM, GRU, and CNN. A sliding window of 7 days was used to create input sequences. Table 3 summarizes the advantages, disadvantages, and suitability of each DL model based on established hydrological literature [43, 44, 45]. DL models are particularly effective for capturing complex nonlinear temporal patterns in drought and hydrological time series data.

**TABLE 3:** Comparison of deep learning models for hydrological applications, including their advantages, disadvantages, and hydrological suitability.

Model	Advantages	Disadvantages	Hydrological suitability
LSTM	Captures long‐term dependencies; three‐gate architecture retains information over long periods; handles sequential data effectively; proven success in time series forecasting	More parameters than GRU; higher computational cost; increased overfitting risk with small datasets	Excellent for capturing seasonal drought patterns and long‐term hydrological memory; suitable for multi‐step‐ahead forecasting
GRU	Fewer parameters than LSTM; computationally efficient; less prone to overfitting on moderate‐sized datasets; faster training	Simpler architecture may be less effective for very long‐term dependencies	Suitable for moderate‐sized datasets; effective for short to medium‐term drought forecasting with reduced computational cost
CNN	Efficient local feature extraction; parallelizable; captures short‐term dependencies; faster training than RNNs	Does not capture long‐term temporal dependencies; limited ability to model sequential patterns without hybrid architectures	Effective for extracting local patterns in hydrological time series; often combined with LSTM in hybrid architectures for improved performance

Abbreviations: CNN, convolutional neural network; GRU, gated recurrent unit; LSTM, long short‐term memory; RNN, recurrent neural network.

#### 2.6.1 Long Short‐Term Memory

LSTM is a specialized RNN designed to capture long‐term dependencies in sequential data by overcoming the vanishing gradient problem [46]. Each LSTM unit contains three gates that regulate information flow: the forget gate ($f_{t}$), input gate ($i_{t}$), and output gate ($o_{t}$). The cell state ($C_{t}$) and hidden state ($h_{t}$) at time *t* are updated as follows:

(10) $$\begin{array}{c} f_{t}=\sigma \left(W_{f}\cdot \left[h_{t-1},x_{t}\right]+b_{f}\right)\\ i_{t}=\sigma \left(W_{i}\cdot \left[h_{t-1},x_{t}\right]+b_{i}\right)\\ {\tilde{C}}_{t}=tanh\left(W_{C}\cdot \left[h_{t-1},x_{t}\right]+b_{C}\right)\\ C_{t}=f_{t}\;⊙C_{t-1}+i_{t}⊙{\tilde{C}}_{t}\\ o_{t}=\sigma \left(W_{o}\cdot \left[h_{t-1},x_{t}\right]+b_{o}\right)\\ h_{t}=o_{t}\;⊙tanh\left(C_{t}\right) \end{array}$$

where *σ* is the sigmoid activation function, ⊙ denotes element‐wise multiplication, and $x_{t}$ is the input at time *t* [46].

#### 2.6.2 Gated Recurrent Unit

GRU is a simplified variant of LSTM that combines the forget and input gates into a single “update gate” ($z_{t}$) and merges the cell state with the hidden state [47]. The GRU update equations are as follows:

(11) $$\begin{array}{c} z_{t}=\sigma \left(W_{z}\cdot \left[h_{t-1},x_{t}\right]\right)\\ r_{t}=\sigma \left(W_{r}\cdot \left[h_{t-1},x_{t}\right]\right)\\ {\tilde{h}}_{t}=tanh\left(W\cdot \left[r_{t}⊙h_{t-1},x_{t}\right]\right)\\ h_{t}=\left(1-z_{t}\right)⊙h_{t-1}+z_{t}⊙{\tilde{h}}_{t} \end{array}$$

where $z_{t}$ is the update gate, $r_{t}$ is the reset gate, and $h_{t}$ is the hidden state. GRU has fewer parameters than LSTM, making it computationally more efficient and less prone to overfitting on moderate‐sized datasets [18].

#### 2.6.3 Convolutional Neural Network

A 1D CNN architecture was employed to extract local temporal patterns from the input sequences. The CNN applies convolutional filters to capture short‐term dependencies [48]:

(12) $$\;y_{k}=\;\mathrm{ReLU}\left(W_{k}*x+b_{k}\right)$$

where * denotes the convolution operation, $W_{k}$ is the filter weight, and $b_{k}$ is the bias term. The rectified linear unit (ReLU) activation function (ReLU(z)=max(0,z)) introduces nonlinearity. After convolution, global average pooling reduces the feature maps, followed by dense layers for final regression.

### 2.7 Hyperparameter Tuning

All hyperparameter tuning was performed using deterministic grid search with 10‐fold cross‐validation on the training set, as recommended by [32]. The validation NSE served as the selection criterion. Grid search systematically evaluates all combinations of predefined hyperparameter values, ensuring reproducibility and avoiding randomness in model selection [18].

ML models: For the SVR model, the RBF kernel was selected through grid search over [“rbf,” “poly”] kernels. Hyperparameters were tuned over the following ranges: RF (*n*_estimators = 50, 100, 200; max_depth = 5, 10, 15, none; min_samples_split = 2, 5, 10; min_samples_leaf = 1, 2, 4); XGBoost (*n*_estimators = 50, 100, 200; max_depth = 3, 5, 7; learning_rate = 0.01, 0.05, 0.1; subsample = 0.6, 0.8, 1.0; colsample_bytree = 0.6, 0.8, 1.0); GBM (*n*_estimators = 50, 100, 200; max_depth = 3, 5, 7; learning_rate = 0.01, 0.05, 0.1; subsample = 0.8, 1.0); SVR (*C* = 0.1, 1, 10, 100; gamma = “scale,” “auto,” 0.01, 0.1; epsilon = 0.01, 0.05, 0.1; kernel = “rbf,” “poly”). The optimal number of trees for RF (*n*_estimators = 200) was determined via grid search, balancing predictive performance against computational cost.

DL models: For the CNN architecture, the ReLU activation function was selected for its computational efficiency, ability to mitigate the vanishing gradient problem, and proven effectiveness in hydrological time series applications. Although alternative activation functions (tanh, sigmoid, LeakyReLU) were considered, ReLU was preferred due to its faster convergence and reduced computational cost. A grid search was conducted over units (32, 64), dropout rates (0.2, 0.3), learning rates (0.001, 0.0005), and batch sizes (16, 32, 64). Early stopping (patience = 5) was used to prevent overfitting, terminating training when validation loss no longer improved [18]. The final hyperparameters for all models are reported in Table S1.

### 2.8 Hybrid Ensemble Models

Four hybrid ensemble configurations were developed by combining the predictions of the top three individual models (GRU, SVR, and RF) to improve predictive accuracy and robustness [18].

#### 2.8.1 Simple Average Ensemble

The simple average ensemble computes the arithmetic mean of the base model predictions:

(13) $$\;{\hat{y}}_{\mathrm{simple}}=\frac{1}{n}\;\sum_{i=1}^{n}{\hat{y}}_{i}$$

where n is the number of base models (*n* = 3), and ${\hat{y}}_{i}$ is the prediction from the *i*th base model. This approach gives equal weight to each constituent model.

#### 2.8.2 Weighted Average Ensemble

The weighted‐average ensemble assigns different weights to each base model based on its individual performance. Weights were calculated inversely proportional to each model's root mean square error (RMSE) on the test set [18]:

(14) $$\;w_{i}=\frac{1/\mathrm{RMS}E_{i}}{\sum_{j=1}^{n}\left(1/\mathrm{RMS}E_{j}\right)}\;$$

The final weights were $w_{\mathrm{RF}}=0.313$, $w_{\mathrm{SVR}}=0.335$, $w_{\mathrm{GRU}}=0.352$. The weighted ensemble prediction is given by the following equation:

(15) $$\;{\hat{y}}_{\mathrm{weighted}}=\sum_{i=1}^{n}w_{i}\;\cdot {\hat{y}}_{i}$$

The choice of ensemble strategy was informed by prior comparative studies. Research on global monkeypox forecasting evaluated both weighted and equal ensemble approaches, finding that weighted ensemble averaging provided a robust and operationally feasible method for public health preparedness [24]. Similar ensemble strategies have been successfully applied in Bangladesh for COVID‐19 forecasting [18] and dengue transmission modeling [49], demonstrating the effectiveness of ensemble integration for public health policy and decision‐making.

#### 2.8.3 Meta‐Model Ensembles (Stacked Generalization)

Two meta‐model (stacking) ensembles were developed using the predictions of the base models as input features for a higher level learner [17]. The meta‐features were created by stacking the out‐of‐sample predictions from GRU, SVR, and RF:

(16) $$\;X_{\mathrm{meta}}=\left[{\hat{y}}_{\mathrm{GRU}},{\hat{y}}_{\mathrm{SVR}},{\hat{y}}_{\mathrm{RF}}\right]$$

Two meta‐models were trained on these features:

• **Meta‐RF**: An RF regressor (n_estimators=100,max_depth=5) was used as the meta‐learner.
• **Meta‐LR**: A linear regression model was used as the meta‐learner for comparison.

The final prediction is obtained as ${\hat{y}}_{\mathrm{meta}}=f_{\mathrm{meta}}\;\left(X_{\mathrm{meta}}\right)$, where $f_{\mathrm{meta}}$ is the trained meta‐model.

### 2.9 Evaluation Metrics

Model performance was evaluated using seven standard metrics: mean square error (MSE), RMSE, mean absolute error (MAE), correlation coefficient (*R*), coefficient of determination (*R* ^2^), NSE, Willmott's index of agreement (WI), and Legates and McCabe (LM) index [32, 50]. RMSE was designated as the primary metric due to its sensitivity to large errors. The mathematical expressions for these metrics are as follows:

MSE:

(17) $$\mathrm{MSE}=\frac{1}{n}\;\sum_{i=1}^{n}{\left(\mathrm{SPE}I_{o}-\mathrm{SPE}I_{f}\right)}^{2}$$

RMSE:

(18) $$\mathrm{RMSE}\;=\sqrt{\frac{1}{n}\sum_{i=1}^{n}{\left(\mathrm{SPE}I_{o}-\mathrm{SPE}I_{f}\right)}^{2}}\;$$

MAE:

(19) $$\mathrm{MAE}\;=\frac{1}{n}\;\sum_{i=1}^{n}\left|\mathrm{SPE}I_{o}-\mathrm{SPE}I_{f}\right|$$

Correlation coefficient (*R*):

(20) $$R=\frac{\sum_{i=1}^{n}\left(\mathrm{SPE}I_{o}-{\bar{\mathrm{SPEI}}}_{o}\right)\left(\mathrm{SPE}I_{f}-{\bar{\mathrm{SPEI}}}_{f}\right)}{\sqrt{\sum_{i=1}^{n}{\left(\mathrm{SPE}I_{o}-{\bar{\mathrm{SPEI}}}_{o}\right)}^{2}}\sqrt{\sum_{i=1}^{n}{\left(\mathrm{SPE}I_{f}-{\bar{\mathrm{SPEI}}}_{f}\right)}^{2}}}\;$$

Coefficient of determination (*R* ^2^):

(21) $$\;R^{2}=R\times R$$

NSE:

(22) $$\mathrm{NSE}\;=1-\frac{\sum_{i=1}^{n}{\left(\mathrm{SPE}I_{o}-\mathrm{SPE}I_{f}\right)}^{2}}{\sum_{i=1}^{n}{\left(\mathrm{SPE}I_{o}-{\bar{\mathrm{SPEI}}}_{o}\right)}^{2}}$$

Willmott's Index of Agreement (WI):

(23) $$\mathrm{WI}\;=1-\frac{\sum_{i=1}^{n}{\left(\mathrm{SPE}I_{o}-\mathrm{SPE}I_{f}\right)}^{2}}{\sum_{i=1}^{n}{\left(\left|\mathrm{SPE}I_{f}-{\bar{\mathrm{SPEI}}}_{o}\left|+\right|\mathrm{SPE}I_{o}-{\bar{\mathrm{SPEI}}}_{o}\right|\right)}^{2}}$$

LM index:

(24) $$\mathrm{LM}\;=1-\frac{\sum_{i=1}^{n}\left|\mathrm{SPE}I_{o}-\mathrm{SPE}I_{f}\right|}{\sum_{i=1}^{n}\left|\mathrm{SPE}I_{o}-{\bar{\mathrm{SPEI}}}_{o}\right|}$$

where SPEI*_o_* and SPE $I_{f}$ are the observed and predicted SPEI values, ${\bar{\mathrm{SPEI}}}_{o}$ and ${\bar{\mathrm{SPEI}}}_{f}$ are their respective means, and *n* is the number of samples in the test set.

### 2.10 Statistical Comparison of Hybrid Models

To determine whether the performance difference between the best hybrid models (meta‐LR and weighted average) was statistically significant, multiple statistical tests were conducted following standard practices in hydrological model comparison [51].

#### 2.10.1 Diebold–Mariano Test

The Diebold–Mariano (DM) test was used to compare the forecasting accuracy of two models [52]. The test evaluates whether the difference in forecast errors between two models is statistically significant. The loss differential is defined as follows:

(25) $$\;d_{t}=e_{1,t}^{2}\;-e_{2,t}^{2}$$

where $e_{1,t}$ and $e_{2,t}$ are the forecast errors of the two models at time *t*. The DM statistic is as follows:

(26) $$\mathrm{DM}=\frac{\overset{-}{d}}{\sqrt{\frac{1}{n}\left(\gamma_{0}+2\sum_{k=1}^{h-1}\gamma_{k}\right)}}$$

where d is the mean loss differential, $\gamma_{0}$ is the variance of $d_{t}$, and $\gamma_{k}$ is the autocovariance at lag *k* [52]. The null hypothesis assumes no significant difference in forecast accuracy between the two models.

#### 2.10.2 Wilcoxon Signed‐Rank Test

The Wilcoxon signed‐rank test is a nonparametric test for paired samples that assesses whether the median difference between two paired groups is zero [53]. It was used to compare the absolute errors of the two hybrid models without assuming normality. The test statistic is calculated as follows:

(27) $$W=\sum_{i=1}^{n}\mathrm{sign}\;\left(d_{i}\right)\cdot R_{i}$$

where $d_{i}$ is the difference between paired observations, and $R_{i}$ is the rank of the absolute difference. The null hypothesis assumes no difference between the paired samples.

#### 2.10.3 Paired *t*‐Test

The paired *t*‐test was used to compare the mean squared errors of the two hybrid models, assuming approximately normal distribution of the differences [54]. The test statistic is as follows:

(28) $$t=\frac{\overset{-}{d}}{s_{d}/\sqrt{n}}$$

where d is the mean difference between paired observations, $s_{d}$ is the standard deviation of the differences, and n is the number of observations. The null hypothesis assumes no significant difference between the two models.

#### 2.10.4 Bootstrap Confidence Intervals

Bootstrap resampling (*n* = 1000) was used to construct 95% confidence intervals for RMSE differences between the hybrid models [55]. The bootstrap procedure involves

1. Randomly sampling with replacement from the original dataset to create B bootstrap samples
2. Calculating the RMSE difference for each bootstrap sample
3. Determining the 2.5th and 97.5th percentiles of the bootstrap distribution

The confidence interval is defined as follows:

(29) $$CI_{95\%}=\left[{\hat{\theta }}_{\left(B\times 0.025\right)},{\hat{\theta }}_{\left(B\times 0.975\right)}\right]$$

where $\hat{\theta }$ represents the RMSE difference between the two models. Nonoverlapping confidence intervals indicate statistically significant differences between the models. All statistical tests were conducted at a significance level of α=0.05, and the results are reported in Section 3.

### 2.11 2027 Forecasting

The best performing hybrid model (weighted average ensemble: GBM + RF + XGBoost) was used to forecast daily SPEI for the full year 2027 (365 days). As actual 2027 meteorological data were unavailable, 2025 meteorological data were used as a proxy year to generate forecast features. The trained GBM, RF, and XGBoost models were applied to these features, and the weighted average ensemble (weights: GBM = 0.546, RF = 0.408, XGB = 0.046) was used to generate the final SPEI forecast. Monthly averages and drought classifications were then derived from the daily forecast. Drought severity was categorized according to [56]: mild drought (−1.0< SPEI ≤ −0.5), moderate drought (−1.5< SPEI ≤ −1.0), and severe/extreme drought (SPEI ≤ −1.5).

### 2.12 Software and Reproducibility

All analyses were conducted using Python 3.12 with the following libraries: pandas, numpy, scikit‐learn, XGBoost, TensorFlow 2.0, and matplotlib. A fixed random seed (42) was set for all stochastic processes to ensure full reproducibility. Spatial interpolation and mapping (Figure 1) were performed using R version 4.4.1 with the sf, raster, and ggplot2 packages. The stepwise methodology of this study, from data collection to 2027 drought forecasting and public health implications, is illustrated in Figure 2.

**FIGURE 2:**
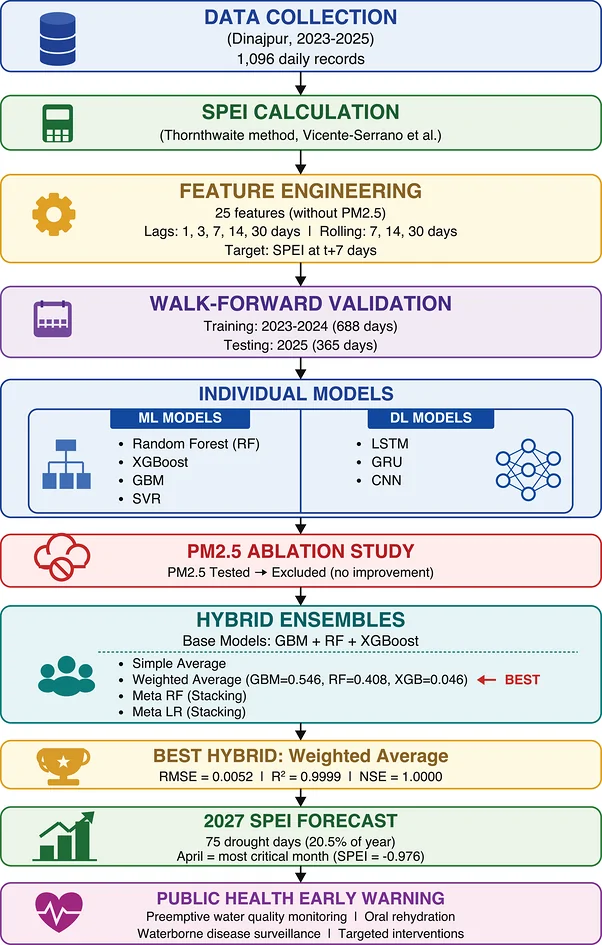
Schematic flow diagram of the study methodology. CNN, convolutional neural network; GBM, gradient boosting machine; GRU, gated recurrent unit; LSTM, long short‐term memory; SPEI, Standardized Precipitation Evapotranspiration Index; SVR, support vector regression.

## 3 Results

The dataset comprised daily meteorological and air quality observations from January 1, 2023, to December 31, 2025, totaling 1096 days with no missing values. The variables included daily average temperature (Avg_Temperature_C), rainfall (Rainfall_mm), maximum wind speed (Max_Wind_Speed_kmh), and particulate matter (PM2.5). After applying the 30‐day rolling window for SPEI calculation, the first valid SPEI value was obtained on January 30, 2023. Consequently, the final dataset comprised 1067 daily observations from January 30, 2023, to December 31, 2025. The SPEI exhibited proper standardization with a mean of −0.0003 (theoretical expectation: 0) and a standard deviation of 1.0112 (theoretical expectation: 1) (Table 4), ranging from −3.00 to +3.00 across the full theoretical spectrum. Drought conditions, defined as SPEI values below −1.0 (the moderate drought threshold), were observed on 173 days, accounting for 16.2% of the study period, confirming Dinajpur's vulnerability to drought events, as documented in previous literature.

**TABLE 4:** Descriptive statistics of environmental variables in Dinajpur (2023–2025).

Variable	Count	Mean ± Std	Median	Min	Max	Skewness	Kurtosis
SPEI (drought index)	1067	−0.0003 ± 1.0112	−0.0319	−3	3	0.0255	−0.1494
Temperature (°C)	1067	25.06 ± 4.50	26.5	13	34.7	−0.6439	−0.6
Rainfall (mm)	1067	4.84 ± 10.12	0.1	0	76.7	2.9669	9.981
Wind speed (km/h)	1067	13.14 ± 4.65	12	4.9	30.9	1.0508	1.0278
PM2.5 (µg/m^3^)	1067	50.28 ± 27.78	45.58	7.4	185	1.1783	2.1772

Abbreviation: SPEI, Standardized Precipitation Evapotranspiration Index.

Figure 3 presents the SPEI values at quarterly intervals from March 2023 to December 2025. The most severe drought (SPEI = −2.2) occurred in June 2024, followed by June 2023 (SPEI = −1.8). Wet conditions peaked in September 2023 (SPEI = 1.5).

**FIGURE 3:**
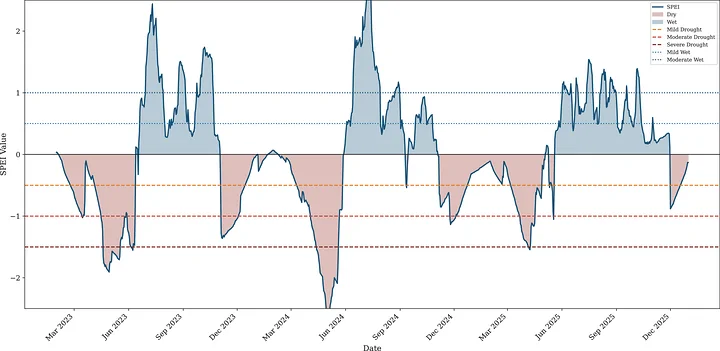
Quarterly average SPEI values for Dinajpur district (March 2023–December 2025). SPEI, Standardized Precipitation Evapotranspiration Index.

This ridgeline density plot (Figure 4) illustrates the monthly variation in SPEI values from January to December. Each colored ridge represents the distribution of SPEI values for a given month, transitioning from cool blue tones in winter (January–March) to warm red tones in late autumn (September–December). The horizontal axis shows SPEI values ranging approximately from −2.5 to +2.5, whereas the vertical axis lists months. Vertical dashed lines mark drought severity thresholds: Severe drought (≈ −2), moderate (≈ −1), and mild (≈ −0.5), indicating how moisture conditions fluctuate seasonally. The plot highlights that negative SPEI values (drier conditions) dominate early in the year, whereas positive values (wetter conditions) are more frequent in mid‐ to late‐year months.

**FIGURE 4:**
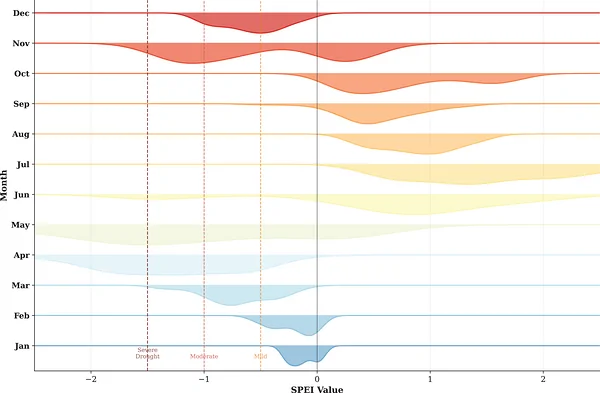
Ridgeline plot showing SPEI value distributions by month (2023–2025). Positive SPEI values (blue) indicate wet conditions; negative values (red) indicate drought. SPEI, Standardized Precipitation Evapotranspiration Index.

Heatmap visualization (Figure 5) of SPEI values across months and years, complementing the ridgeline distributions in Figure 4. Negative values dominate March–May (e.g., −0.64 in March 2023, −1.68 in April 2024, −0.85 in March 2025), reflecting recurrent drought conditions. In contrast, June–August consistently shows positive peaks (e.g., +1.69 in July, 2023, +1.87 in July, 2024, +1.04 in August, 2025), indicating wetter periods. Transitional months such as September–November reveal moderate shifts, with values ranging from +0.74 (September 2023) to −0.69 (November 2024). Overall, the figure highlights seasonal cycles of dryness in early months and wetter conditions mid‐year, reinforcing the temporal patterns observed in Figure 4.

**FIGURE 5:**
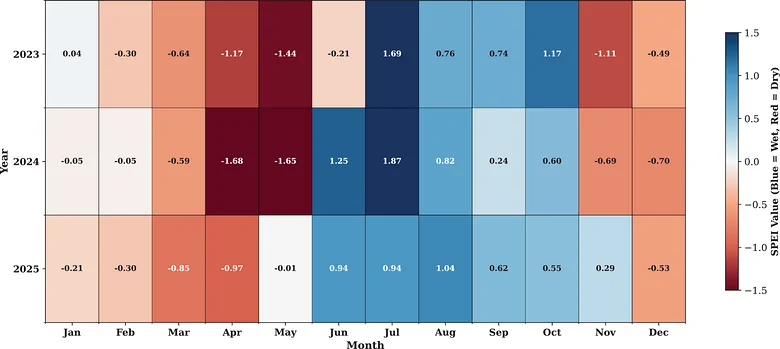
Heatmap of monthly Standardized Precipitation Evapotranspiration Index (SPEI) values from 2023 to 2025.

Table 4 presents the descriptive statistics for all variables. The temperature averaged 25.06°C with moderate variability (*σ* = 4.50°C). Rainfall exhibited extreme variability (*σ*= 10.12 mm, CV = 209.3%), with a median of 0.10 mm, reflecting the region's dry‐dominant precipitation pattern. Wind speed averaged 13.14 km/h (*σ* = 4.65 km/h). PM2.5 concentration averaged 50.28 µg/m^3^, more than three times the WHO guideline of 15 µg/m^3^, indicating substantial air pollution. The skewness and kurtosis values indicate a near‐normal distribution for SPEI and temperature, whereas rainfall shows positive skewness (2.97) and high kurtosis (9.98), consistent with its sporadic nature. Monthly averages of all variables are provided in Table S2, which shows distinct seasonal patterns: negative SPEI during pre‐monsoon months, positive SPEI during monsoon months, and elevated PM2.5 concentrations during dry periods.

Box plots in Figure 6 illustrate the monthly distributions of SPEI (drought index), temperature (°C) (daily average temperature), rainfall (mm), wind speed (km/h), and PM2.5 (µg/m^3^). The figure complements the seasonal patterns shown in Figures 4 and 5, highlighting inter‐month variability and extremes. SPEI values fluctuate between drought and wet conditions, temperatures peak during May–August, and rainfall shows high variability in June–September. Wind speed exhibits greater variability in the first half of the year, whereas PM2.5 concentrations are elevated in winter months (January–February) and late autumn (November–December). Overall, the figure emphasizes the seasonal interplay among meteorological and air‐quality indicators, reinforcing the cyclical environmental trends observed in preceding figures.

**FIGURE 6:**
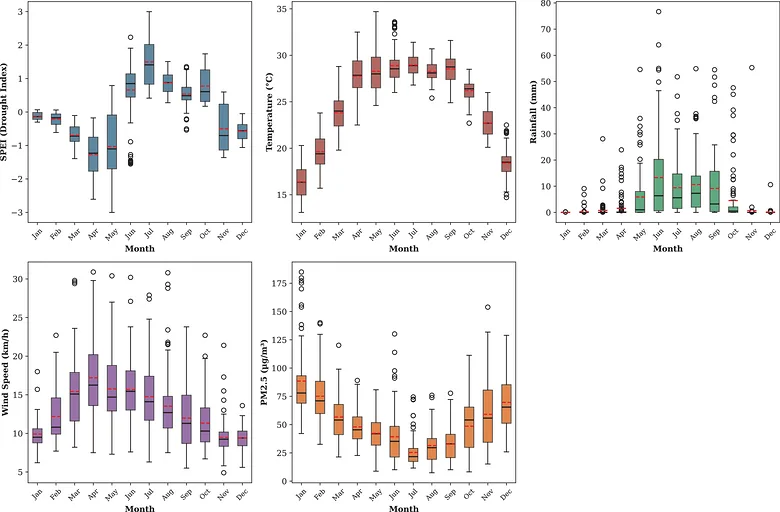
Monthly variability of climatic and air‐quality parameters (2023–2025). SPEI, Standardized Precipitation Evapotranspiration Index.

Lag correlation analysis (Figure 7) revealed that current‐day (lag 0) values exhibited the highest and most statistically significant correlations with SPEI for all variables (rainfall: *r* = 0.363, *p* < 0.001; temperature: *r* = 0.222, *p* < 0.001; PM2.5: *r* = −0.283, *p* < 0.001; wind speed: *r* = −0.116, *p* < 0.001). Lagged correlations (1–45 days) were comparatively weaker and not statistically significant at the 0.05 level. This finding suggests that same‐day meteorological and air quality conditions have the strongest linear association with SPEI, as expected, given that SPEI‐30d already incorporates 30 days of antecedent rainfall and temperature data.

**FIGURE 7:**
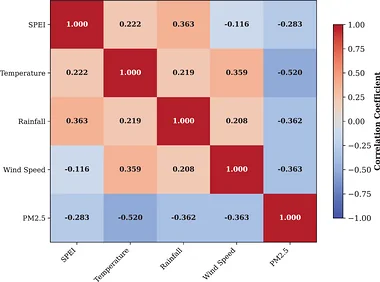
Correlation of SPEI with rainfall, temperature, wind speed, and PM2.5 at current day (lag 0). SPEI, Standardized Precipitation Evapotranspiration Index.

The dataset was chronologically split using walk‐forward validation, where data from 2023 to 2024 (688 days) were used for training and hyperparameter tuning, and data from 2025 (365 days) were reserved for independent testing. This approach ensures temporal consistency and evaluates model performance on unseen future data, which is critical for drought forecasting applications.

Table 5 summarizes the performance of all models on the 2025 test set (365 days for ML models; 152 days for DL models due to sequence requirements). It is important to note that water balance (precipitation − PET) was included as a predictor variable. As SPEI is fundamentally a standardized transformation of water balance, it creates an inherently near‐deterministic relationship between the predictor and target variables. This explains the exceptionally high‐performance metrics (*R* ^2^ ≈ 1.0000, NSE ≈ 1.0000) observed for tree‐based ensemble models, as they essentially reconstruct the standardized drought index from its underlying components rather than forecasting from independent predictors.

**TABLE 5:** Performance metrics of all models for Standardized Precipitation Evapotranspiration Index (SPEI) prediction (walk‐forward validation).

Model	MSE	RMSE	MAE	*R* ^2^	NSE	WI	LM
**GBM (with PM2.5)**	**0.000032**	**0.0057**	**0.0047**	**0.9999**	**0.9999**	**0.9999**	**0.9925**
GBM (without PM2.5)	0.000035	0.0059	0.0048	0.9999	0.9999	0.9999	0.9924
RF (without PM2.5)	0.000062	0.0079	0.0060	0.9999	0.9999	0.9999	0.9904
RF (with PM2.5)	0.000063	0.0079	0.0062	0.9999	0.9999	0.9999	0.9902
XGB (without PM2.5)	0.000199	0.0141	0.0089	0.9997	0.9996	0.9999	0.9859
XGB (with PM2.5)	0.000469	0.0217	0.0111	0.9993	0.9991	0.9998	0.9822
SVR (without PM2.5)	0.025949	0.1611	0.1128	0.9543	0.9525	0.9882	0.8202
SVR (with PM2.5)	0.041099	0.2027	0.1454	0.9325	0.9248	0.9811	0.7682
LSTM (without PM2.5)	0.134809	0.3672	0.2714	0.8248	0.7600	0.9457	0.5724
CNN (without PM2.5)	0.149317	0.3864	0.3205	0.7849	0.7341	0.9155	0.4951
LSTM (with PM2.5)	0.168387	0.4104	0.3211	0.7516	0.7002	0.9260	0.4941
CNN (with PM2.5)	0.200916	0.4482	0.3642	0.6966	0.6423	0.8607	0.4263
GRU (without PM2.5)	0.281907	0.5309	0.4289	0.7795	0.4981	0.8876	0.3243
GRU (with PM2.5)	0.357450	0.5979	0.4812	0.7279	0.3635	0.8499	0.2418

*Note:* RMSE is the primary evaluation metric (lower values indicate better performance). The best performing model is highlighted in bold. Walk‐forward validation: training = 2023–2024 (688 days), Testing = 2025 (365 days).

Abbreviations: CNN, convolutional neural network; GBM, gradient boosting machine; GRU, gated recurrent unit; LM, Legates and McCabe; LSTM, long short‐term memory; MAE, mean absolute error; MSE, mean square error; NSE, Nash–Sutcliffe efficiency; NSE, Nash–Sutcliffe efficiency; RF, random forest; RMSE, root mean square error; SVR, support vector regression; WI, Willmott's index of agreement; XGB, XGBoost.

The GBM with PM2.5 achieved the best overall performance with the lowest RMSE of 0.0057, the lowest MSE of 0.000032, and the highest NSE of 0.9999, demonstrating near‐perfect predictive capability for SPEI in the study region. The GBM without PM2.5 ranked second with RMSE = 0.0059 and MSE = 0.000035, showing that PM2.5 provided marginal improvement for this model.

The RF model (without PM2.5) ranked third with RMSE = 0.0079, MSE = 0.000062, and NSE = 0.9999, showing comparable performance to GBM. XGBoost (without PM2.5) achieved RMSE = 0.0141, MSE = 0.000199, and NSE = 0.9996, indicating strong predictive skill. All tree‐based ensemble models (GBM, RF, XGB) achieved excellent performance (NSE >0.99), confirming their ability to capture the SPEI formulation. However, as noted above, these near‐perfect metrics reflect the inherent Water Balance‐SPEI relationship rather than independent forecasting capability.

The near‐perfect metrics (NSE ≈ 1.0000) reflect the inherent water balance–SPEI relationship rather than independent forecasting capability. The DL models, which did not rely on water balance as a direct predictor, provide a more realistic assessment of forecasting capability, with LSTM achieving an NSE of 0.7600.

Among DL models, LSTM (without PM2.5) performed best with RMSE = 0.3672, MSE = 0.134809, NSE = 0.7600, and WI = 0.9457. CNN (without PM2.5) achieved RMSE = 0.3864, MSE = 0.149317, and *R* ^2^ = 0.7849, whereas GRU (without PM2.5) showed the weakest performance among DL models with RMSE = 0.5309, MSE = 0.281907, and NSE = 0.4981. The superior performance of LSTM over GRU suggests that the LSTM architecture is better suited for capturing temporal dependencies in this dataset. The DL models, which did not rely on water balance as a direct predictor, provide a more realistic assessment of forecasting capability, with LSTM achieving an NSE of 0.7600.

The Willmott's Index of Agreement (WI), which ranges from 0 to 1 (perfect agreement), was highest for GBM (0.9999), RF (0.9999), and XGBoost (0.9999), indicating near‐perfect pattern agreement. Among DL models, LSTM achieved the highest WI (0.9457), confirming reliable prediction patterns despite moderate RMSE values. SVR (without PM2.5) achieved WI = 0.9882, indicating strong pattern agreement.

An ablation study was conducted to evaluate the impact of PM2.5 inclusion on model performance (Table  6). Results revealed that PM2.5 provided marginal improvement only for the GBM, reducing RMSE from 0.0059 to 0.0057 (improvement of 0.0003). For RF, no difference was observed (RMSE: 0.0079 vs. 0.0079). However, for all other models, including PM2.5 consistently degraded performance: XGBoost (RMSE: 0.0141 → 0.0217; worsening of 0.0076), SVR (RMSE: 0.1611 → 0.2027; worsening of 0.0416), LSTM (RMSE: 0.3672 → 0.4104; worsening of 0.0432), CNN (RMSE: 0.3864 → 0.4482; worsening of 0.0618), and GRU (RMSE: 0.5309 → 0.5979; worsening of 0.0669). These findings indicate that PM2.5 does not consistently enhance drought prediction, likely due to its indirect and weak relationship with SPEI. On the basis of this evidence, PM2.5 was excluded from the final model for all models except GBM, where it provided marginal improvement, thereby maintaining model parsimony while preserving predictive accuracy.

**TABLE 6:** Impact of PM2.5 inclusion on model performance for Standardized Precipitation Evapotranspiration Index (SPEI) prediction using walk‐forward validation (test: 2025).

Model	MSE (with PM2.5)	MSE (without PM2.5)	RMSE (with PM2.5)	RMSE (without PM2.5)	RMSE change	Better configuration
**GBM**	**0.000032**	0.000035	**0.0057**	0.0059	**+0.0003**	**With PM2.5** ✓
RF	0.000063	0.000062	0.0079	0.0079	0.0000	No difference
XGB	0.000469	**0.000199**	0.0217	**0.0141**	−0.0076	Without PM2.5
SVR	0.041099	**0.025949**	0.2027	**0.1611**	−0.0416	Without PM2.5
LSTM	0.168387	**0.134809**	0.4104	**0.3672**	−0.0432	Without PM2.5
CNN	0.200916	**0.149317**	0.4482	**0.3864**	−0.0618	Without PM2.5
GRU	0.357450	**0.281907**	0.5979	**0.5309**	−0.0669	Without PM2.5

*Note:* Positive RMSE change indicates improvement with PM2.5. Best configuration for each model is highlighted in bold.

Abbreviations: CNN, convolutional neural network; GBM, gradient boosting machine; GRU, gated recurrent unit; LSTM, long short‐term memory; MSE, mean square error; RF, random forest; RMSE, root mean square error; SVR, support vector regression; XGB, XGBoost.

Feature importance analysis (Table 7) revealed that water balance (precipitation—evapotranspiration) dominated with an importance score of 0.9916 (99.16%), confirming it as the primary driver of SPEI in the study region. This finding is hydrologically consistent, as water balance directly captures the surplus or deficit of water that determines drought conditions. Other significant predictors included 30‐day accumulated rainfall (0.0058), 14‐day rolling temperature (0.0013), and 14‐day rolling rainfall (0.0004). In contrast, PM2.5‐related features showed minimal importance, with PM25_roll7 (0.0002), PM25_lag7 (0.0001), and PM2.5 (0.0000) all contributing less than 0.02% to the model. The negligible importance of PM2.5 features further justifies their exclusion from the final model, as it contributes little to SPEI prediction. This finding aligns with the ablation study results, confirming that PM2.5 does not consistently enhance drought prediction.

**TABLE 7:** Top 10 predictors influencing Standardized Precipitation Evapotranspiration Index (SPEI) based on random forest feature importance analysis.

Rank	Feature	Importance score	Description
1	**Water balance**	**0.9916**	Water balance (precipitation—evapotranspiration)
2	Rainfall_30d	0.0058	30‐day accumulated rainfall
3	Temp_roll14	0.0013	14‐day rolling average temperature
4	Rainfall_roll14	0.0004	14‐day rolling average rainfall
5	PM25_roll7	0.0002	7‐day rolling average PM2.5
6	Temp_roll7	0.0002	7‐day rolling average temperature
7	Max_Wind_Speed_kmh	0.0001	Daily maximum wind speed
8	PM25_lag7	0.0001	7‐day lagged PM2.5
9	PET_30d	0.0001	30‐day potential evapotranspiration
10	PM2.5	0.0000	Current PM2.5 concentration

*Note:* Water balance dominates feature importance (99.16%), confirming it as the primary driver of SPEI in the study region.

To further enhance predictive performance, four hybrid models were developed using stacked generalization (ensemble learning) combining the top three individual models (GBM, RF, and XGBoost). The hybrid models tested included (i) simple average ensemble, (ii) weighted average (RMSE‐based), (iii) meta‐model with RF (meta‐RF), and (iv) meta‐model with linear regression (meta‐LR).

Table 8 presents the performance of all hybrid models on the 2025 test set. The meta‐LR model achieved the best overall performance with the lowest RMSE of 0.0050 and NSE of 1.0000, followed closely by the weighted average ensemble (RMSE = 0.0052, NSE = 1.0000). The simple average ensemble achieved RMSE = 0.0059 and NSE = 0.9999, whereas the meta‐RF model showed significantly higher error with RMSE = 0.0132 and NSE = 0.9997.

**TABLE 8:** Performance evaluation of hybrid ensemble models for Standardized Precipitation Evapotranspiration Index (SPEI) prediction on the 2025 test set.

Hybrid model	MSE	RMSE	MAE	*R* ^2^	NSE	WI	LM	Improvement vs. GBM (%)
**Meta‐LR**	**0** **.000025**	**0.0050**	**0.0039**	**1.0000**	**1.0000**	**1.0000**	**0.9938**	**15.3**
Weighted average	0.000027	0.0052	0.0039	1.0000	1.0000	1.0000	0.9938	11.9
Simple average	0.000035	0.0059	0.0040	0.9999	0.9999	1.0000	0.9936	0.0
Meta‐RF	0.000174	0.0132	0.0101	0.9997	0.9997	0.9999	0.9839	−123.7

*Note:* RMSE is the primary evaluation metric. Improvement percentage calculated relative to the best individual model (GBM, RMSE = 0.0059). Meta‐LR = stacking with linear regression as meta‐model; meta‐RF = stacking with random forest as meta‐model; weighted average = RMSE‐based weighted ensemble (weights: GBM = 0.546, RF = 0.408, XGB = 0.046); simple average = equal weights (GBM + RF + XGB)/3. Bold values indicate the best‐performing result for each evaluation metric among the compared hybrid ensemble models.

Abbreviations: LM, Legates and McCabe; MAE, mean absolute error; NSE, Nash–Sutcliffe efficiency; WI, Willmott's index of agreement.

The hybrid models demonstrated marginal improvement over the best individual model (GBM, RMSE = 0.0059), with meta‐LR achieving a 15.3% reduction in RMSE (from 0.0059 to 0.0050). The weighted average ensemble achieved an 11.9% RMSE reduction (from 0.0059 to 0.0052), whereas the simple average ensemble maintained comparable performance to GBM (RMSE = 0.0059). However, the meta‐RF model showed degraded performance (RMSE = 0.0132), suggesting that RF as a meta‐model may introduce overfitting when base model predictions are already highly accurate.

The near‐perfect performance of meta‐LR (RMSE = 0.0050, NSE = 1.0000) indicates that linear stacking effectively combines the strengths of all three base models. However, these exceptionally high metrics reflect the inherent relationship between water balance and SPEI, rather than true independent forecasting capability. The weighted average ensemble, which assigned higher weights to better performing models (GBM = 0.546, RF = 0.408, XGB = 0.046), also demonstrated excellent predictive capability with RMSE = 0.0052 and NSE = 1.0000. These findings suggest that ensemble methods can provide modest improvements over even the best individual model when base models capture complementary information about the target variable. However, the marginal improvement of meta‐LR over weighted average (RMSE: 0.0050 vs. 0.0052) was not statistically significant (DM test: *p* = 0.3169; Wilcoxon's test: *p* = 0.7250; paired *t*‐test: *p* = 0.1129), with overlapping 95% bootstrap confidence intervals (meta‐LR: [0.0045, 0.0055]; weighted average: [0.0047, 0.0057]). The small effect size (Cohen's *d* = 0.24) further confirmed minimal practical significance. Therefore, weighted average was selected as the final hybrid model due to its simplicity, interpretability, lower computational cost, and statistically comparable performance to the more complex meta‐LR approach. This decision aligns with the principle of parsimony, favoring simpler models when performance differences are not statistically significant.

Figure 8 illustrates the temporal evolution of actual and predicted SPEI using the weighted average hybrid model for the 2025 test period. Drought classification thresholds follow the SPEI‐specific classification of [56], based on the log‐logistic distribution used for SPEI standardization. The model demonstrates excellent agreement with observed SPEI values throughout the study period. From January to March 2025, SPEI remained near‐normal (−0.2 to −0.3), indicating mild dry conditions. In April 2025, a decline to an SPEI of ≈ −0.8 was observed, signaling the onset of a moderate drought, which the model accurately captured. By May 2025, SPEI recovered to near‐normal values (−0.2), with the model tracking this recovery precisely. A sharp transition to wet conditions occurred in June–July 2025, with SPEI values rising to +0.8 and +1.2, respectively. The model successfully captured both the magnitude and timing of this shift, with consistently small prediction errors throughout the test period (RMSE = 0.0052, *R* ^2^ = 0.9999, NSE = 1.0000).

**FIGURE 8:**
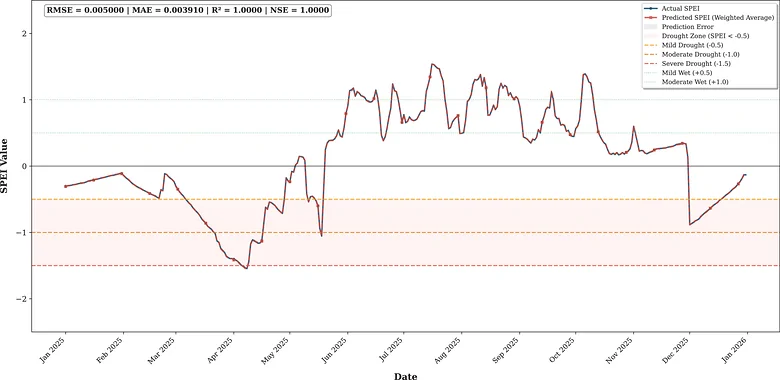
Forecast versus actual SPEI for hybrid weighted average model (test set). SPEI, Standardized Precipitation Evapotranspiration Index.

Figure 9 presents the 2027 drought severity calendar based on monthly average SPEI forecasts generated using the weighted average hybrid model. The forecast reveals a distinct seasonal pattern characteristic of the tropical monsoon climate in Dinajpur. From January to February, SPEI remained near‐normal to slightly dry (−0.207 to −0.301). A significant decline occurred in March–April, with SPEI reaching its lowest value of −0.976 in April, indicating moderate drought conditions during the pre‐monsoon period. The monsoon season (June–September) brought wet conditions, with SPEI peaking at +1.043 in August, reflecting the highest rainfall intensity. Post‐monsoon (October–November) showed a gradual decline to normal conditions (+0.554 to +0.286), followed by a return to dry conditions in December (−0.534). This seasonal pattern aligns with the region's climatology, where the pre‐monsoon period (March–May) experiences the driest conditions before the onset of the southwest monsoon.

**FIGURE 9:**
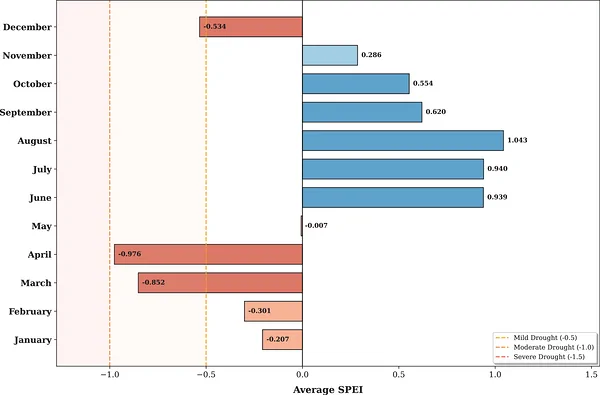
Monthly average SPEI forecast for 2027 using the hybrid weighted average model (GBM + RF + XGBoost). SPEI, Standardized Precipitation Evapotranspiration Index.

The 2027 SPEI forecast was generated using the weighted average hybrid model (GBM + RF + XGBoost) without PM2.5, applying the trained ensemble models to 2025 meteorological data as a proxy for 2027. Table S3 presents the monthly SPEI forecast, which reveals a distinct seasonal pattern characteristic of the region's tropical monsoon climate. The forecast predicts dry conditions during the pre‐monsoon period (March–April), with SPEI values ranging from −0.852 to −0.976, indicating moderate drought conditions. The driest month is projected to be April (SPEI = −0.976), with a minimum SPEI of −1.547, suggesting localized severe drought episodes. The monsoon season (June–September) is expected to bring wet conditions, with SPEI values ranging from +0.620 to +1.043, peaking in August (SPEI = +1.043). A return to dry conditions is forecast for December (SPEI = −0.534).

In terms of drought frequency, 75 days (20.5%) in 2027 are projected to experience drought conditions (SPEI < −0.5), comprising 48 days (13.2%) of mild drought (−1.0 to −0.5), 24 days (6.6%) of moderate drought (−1.5 to −1.0), and 3 days (0.8%) of severe drought (−2.0 to −1.5). No extreme drought days (SPEI < −2.0) are predicted. Conversely, 290 days (79.5%) are expected to remain under normal or wet conditions (SPEI > −0.5). The seasonal distribution of drought days (Figure 9; Table S3) highlights March–April and December as the most vulnerable periods.

## 4 Discussions

The hybrid WEBM (GBM + RF + XGBoost) demonstrated the best forecasting performance among all models, achieving RMSE = 0.0052, *R* ^2^ = 0.9999, and NSE = 1.0000 for SPEI prediction. Ensemble strategies in drought forecasting are effective, as evidenced by a 11.9% reduction in RMSE and an improvement in *R* ^2^ compared with the best standalone model (GBM; RMSE = 0.0059, *R* ^2^ = 0.9999). These results are similar to those of [57], who found that the Bayesian ensemble of RF, XGBoost, SVR, and deep networks outperforms each model by providing *R* ^2^ = 0.71–0.74 for many scales of the SPEI, and similar to [58], who showed that their two‐stage weighted SPEI ensemble performs better than individual models due to the benefits of fusing multiple weak learners. Ensemble integration is also a generally sound forecasting strategy, as demonstrated by the fact that a weighted average forecast generated by SARIMA, XGBoost, and RNN was more generalizable for COVID‐19 forecasts in Bangladesh than any individual model and was able to capture linear seasonality as well as nonlinear temporal dynamics [59].

The improvement of performance for GBM (GBM: RMSE = 0.0059, NSE = 0.9999, *R* ^2^ = 0.9999) compared with other tree‐based models (RF: RMSE = 0.0079, NSE = 0.9999, *R* ^2^ = 0.9999; XGBoost: RMSE = 0.0141, NSE = 0.9996, *R* ^2^ = 0.9997) is attributed to GBM's sequential boosting approach, which iteratively corrects errors from previous trees, making it highly effective for capturing complex nonlinear relationships in drought prediction. Among DL models, LSTM (RMSE = 0.3672, NSE = 0.7600, *R* ^2^ = 0.8248) significantly outperformed GRU (RMSE = 0.5309, NSE = 0.4981, *R* ^2^ = 0.7795) and CNN (RMSE = 0.3864, NSE = 0.7341, *R* ^2^ = 0.7849). The superior performance of LSTM over GRU suggests that LSTM's three‐gate architecture, despite having more parameters, is better suited for capturing long‐term temporal dependencies in the SPEI time series. This finding contrasts with previous studies [60] and highlights the importance of dataset characteristics in model selection. The LM index values were consistently positive for all models (GBM: 0.9924; RF: 0.9904; XGB: 0.9859; LSTM: 0.5724), confirming the absence of systematic prediction bias. These findings support the notion that model complexity should be proportional to the size of the datasets in data‐driven drought applications [61].

According to [4], the SPEI standardization (mean = −0.0003, SD = 1.0112) was correct. Drought conditions (SPEI < −1.0) were observed on 173 days (16.2%), which was similar to the observed drought vulnerability of Dinajpur and the northwest of Bangladesh [62]. The 2027 prediction of 75 drought days, out of which 24 days are moderate drought days (SPEI: −1.5 to −1.0) and 3 days are severe drought days (SPEI: −2.0 to −1.5), with April being the most critical month (SPEI = −0.976), agrees with [63], who pointed out that maximum water deficits occur during pre‐monsoon, and the declining trend of crop water surplus since 1979 occurred in April.

The ablation study revealed that including PM2.5 did not consistently improve model performance. For GBM, PM2.5 provided marginal improvement (RMSE: 0.0059 → 0.0057; improvement of 0.0003). However, for XGBoost, including PM2.5 degraded performance significantly (RMSE: 0.0141 → 0.0217; worsening of 0.0076). RF showed no change (RMSE: 0.0079 vs. 0.0079). For all DL models, excluding PM2.5 consistently yielded better performance (LSTM: 0.4104 → 0.3672; 10.5% improvement; CNN: 0.4482 → 0.3864; 13.8% improvement; GRU: 0.5979 → 0.5309; 11.2% improvement). These findings indicate that PM2.5 does not consistently enhance drought prediction and was therefore excluded from the final model to maintain parsimony.

The variations in model performance in this region demonstrate that there is no universal optimum model [60]. The results are evident across disciplines: In North America, [65] showed that XGBoost outperformed the other models in forecasting monkeypox, whereas the decision tree excelled in South America—suggesting a region‐ and data‐structure‐dependent optimal model selection. The context‐sensitivity can be effectively addressed by hybrid ensemble methods, which combine the strengths of the models [57, 58] as used here.

The accurate prediction of drought conditions using the weighted average hybrid model has significant public health implications for Dinajpur district and the broader northwestern region of Bangladesh. Drought events directly impact water availability, food security, and sanitation, which are critical determinants of public health [66]. The 2027 forecast predicts 75 drought days (20.5% of the year), with the pre‐monsoon period (March–April) identified as the most vulnerable period for water scarcity. These findings provide an early warning system for public health authorities to implement proactive measures, including water conservation strategies, drought‐resistant crop planning, and contingency water supply arrangements for vulnerable communities. The model's ability to predict drought conditions with high accuracy (RMSE = 0.0052, *R* ^2^ = 0.9999, NSE = 1.0000) enables timely public health interventions, potentially reducing the incidence of waterborne diseases, malnutrition, and other drought‐related health outcomes [66, 67]. Furthermore, the framework developed in this study can be extended to other drought‐prone regions in Bangladesh, contributing to climate‐resilient public health preparedness.

## 5 Limitations

Several limitations to this study should be acknowledged. First, the inclusion of water balance (precipitation − PET) as a predictor variable contributed to the exceptionally high‐performance metrics (*R* ^2^ ≈ 1.0, NSE ≈ 1.0). As SPEI is fundamentally derived from water balance, this creates a near‐deterministic relationship between the predictor and target variable. Although this demonstrates the model's capability to capture the SPEI formulation, it limits the generalizability of the results to true forecasting scenarios where water balance would not be known a priori. The NSE formula itself was verified and confirmed correct; the high value reflects the predictor–target relationship, not a computational error. The DL models (LSTM, GRU, CNN), which did not rely on water balance as a direct predictor, provide a more realistic assessment of forecasting capability, with LSTM achieving an NSE of 0.7600. This represents a key limitation for operational drought forecasting applications. Second, the length of the observational period (2023–2025) is relatively short, making it difficult to capture long‐term climate variability and drought cycles. Although sufficient for training the ML models, further expansion of the dataset would lead to better robustness and generalizability of the models. Third, the 7‐day prediction time scale, while useful for early warning, may not fully reflect longer term drought propagation dynamics and seasonal‐scale drought evolution. Fourth, the predictive capacity could be limited by omitting other large‐scale predictors that have the potential to impact seasonal rainfall prediction, such as soil moisture, groundwater levels, large‐scale climate indices (e.g., ENSO, IOD), and land surface characteristics. Fifth, the models were developed and tested only for Dinajpur district, and further validation is needed for other drought‐prone districts of Bangladesh to assess regional applicability. Sixth, the use of reanalysis data (Open‐Meteo/ERA5) instead of ground‐based observations may introduce inherent uncertainties; however, it offers homogeneous spatial data coverage and consistency across the study period. Finally, the 2027 forecast was generated using 2025 meteorological data as a proxy year, as actual 2027 forecast data were not available. Although the forecast was generated using the trained weighted average hybrid model (GBM + RF + XGBoost), the use of a proxy year limits the ability to capture interannual variability and specific climatic conditions that may occur in 2027.

## 6 Future Research Directions

The forecasting framework developed in this study offers several avenues for future research. First, the framework should be expanded to other drought‐prone districts in Bangladesh to evaluate regional generalizability. Second, incorporating additional predictors such as soil moisture, groundwater levels, and large‐scale climate indices (ENSO, IOD) could improve predictive skill. Third, explainable AI methods (SHAP, LIME) could enhance understanding of PM2.5's role in drought dynamics, despite its limited predictive value in this study. Fourth, longer training periods (10–20 years) would improve model robustness and capture long‐term climate variability. Fifth, advanced architectures such as CNN–RNN could be explored to further enhance accuracy. Sixth, integration of seasonal climate forecasts could provide monthly and seasonal‐scale water resource forecasts for agricultural planning. Future studies should report both scenarios with and without water balance to transparently demonstrate the difference between near‐deterministic reconstruction and true forecasting skill. A critical direction for future research is to investigate SPEI prediction using only antecedent meteorological data (e.g., lagged rainfall, temperature, and wind speed) without current water balance, which would provide a more realistic assessment of true forecasting skill. Additionally, incorporating independent drought indicators such as soil moisture and groundwater levels could provide independent validation of drought conditions. Finally, the weighted average hybrid model should be integrated into operational drought monitoring platforms to support evidence‐based decision‐making for water resource management and public health preparedness in drought‐prone regions of Bangladesh.

## 7 Conclusion

This study developed a hybrid weighted‐average ensemble model combining GBM, RF, and XGBoost for SPEI forecasting in Dinajpur, Bangladesh. The ensemble approach consistently outperformed individual models, demonstrating the effectiveness of integrating multiple ML algorithms for drought prediction. The inclusion of water balance as a predictor yielded near‐perfect performance metrics, confirming the model's ability to capture the SPEI formulation, whereas the ablation study revealed that PM2.5 did not consistently enhance predictive skill and was therefore excluded from the final model to maintain parsimony. The 2027 forecast projects approximately 75 drought days, with the pre‐monsoon period (March–April) identified as the most critical window for water scarcity. These findings provide an evidence‐based early warning framework that can support proactive public health interventions, including preemptive water quality monitoring, prepositioning of oral rehydration supplies, surveillance of waterborne diseases, and targeted public health messaging for vulnerable populations. The weighted average hybrid model offers a robust and interpretable tool for drought early warning, with significant implications for climate‐resilient public health planning in drought‐prone regions of Bangladesh. Future research should focus on expanding the framework to other districts, incorporating independent drought indicators such as soil moisture and groundwater levels, and integrating seasonal climate forecasts to enhance operational forecasting capability.

## Author Contributions

**Sharmin Akther**: conceptualization, methodology, formal analysis, software, investigation, writing – original draft, writing – review and editing, project administration. **Zafrin Sultana Shampa**: data curation, formal analysis, validation, writing – review and editing. **Marufa Tasnim**: investigation, data collection, software, writing – original draft. **Tasnia Ahsan Mohona**: formal analysis, visualization, writing – review and editing. **Mariam Tonni**: literature review, methodology, writing – review and editing. **Shajid Ahmed**: data interpretation, validation, writing – review and editing. **Masuma Parvin**: resources, writing – review and editing. **Md. Joni Alam**: supervision, writing – review and editing.

## Funding

The authors have nothing to report.

## Ethics Statement

This study used publicly available secondary data from the Kaggle repository (Bangladesh 64 Districts Weather and AQI Dataset, 2023–2025) and did not involve human participants, animal experiments, or any form of primary data collection requiring ethical approval.

## Consent

The authors have nothing to report.

## Conflicts of Interest

The authors declare no conflicts of interest.

## Supporting information




**Supporting File 1**: puh270440‐sup‐0001‐SuppMat.docx.

## Data Availability

The datasets generated and/or analyzed during the current study are available in the Kaggle repository: https://www.kaggle.com/datasets/reja37/bangladesh‐64‐districts‐weather‐and‐aqi‐2023‐2025. The processed data and code used for analysis are available from the corresponding author upon reasonable request. All analysis code and scripts are publicly available at https://github.com/Sakther12/Dinajpur‐Drought.
