# Supplementary material for: Integrating Meteorological Data for Drought Prediction: A Hybrid Ensemble Machine Learning Approach in Northwestern Bangladesh
Source: Public Health Chall. 2026 Nov 7;5(4):e70440. doi: 10.1002/puh2.70440 (PMC13744606; doi:10.1002/puh2.70440)
Supplement: Supplementary file 1 [file PUH2-5-e70440-s001.docx]

**Figure S1:** Monthly variations in (a) precipitation and (b) PM2.5 concentrations across Dinajpur District (2023–2025). The figure clearly illustrates the inverse relationship between rainfall and PM2.5, with monsoon months showing peak precipitation and lowest PM2.5 concentrations, while winter months exhibit minimal rainfall and maximum PM2.5 levels. The WHO guideline of 25 µg/m³ is indicated for reference.

Table S1 presents the optimal hyperparameters for all machine learning and deep learning models, obtained via a deterministic grid search with 10-fold cross-validation. For Random Forest, the optimal configuration was n_estimators = 200, max_depth = 20, and min_samples_split = 2. XGBoost performed best with n_estimators = 100, max_depth = 7, learning_rate = 0.1, and subsample = 1.0. Gradient Boosting achieved optimal performance with n_estimators = 200, max_depth = 5, learning_rate = 0.05, and subsample = 0.8. SVR's optimal parameters were C = 10, gamma = 'scale', and epsilon = 0.05. For deep learning models, all architectures (LSTM, GRU, CNN) used learning rate = 0.001, batch size = 32, dropout = 0.2, and 20 epochs with early stopping patience = 5. LSTM and GRU utilized 64 units in the first layer and 32 units in the second layer, while CNN employed 64 filters with kernel size = 3.

**Table S1:** Optimal hyperparameters for all seven models (Random Forest, XGBoost, Gradient Boosting, SVR, LSTM, GRU, and CNN) were identified through deterministic grid search with 10-fold cross-validation.

| **Model** | **Type** | **Hyperparameters** |
| --- | --- | --- |
| **Random Forest** | ML | max_depth = 20, min_samples_split = 2, n_estimators = 200 |
| **XGBoost** | ML | learning_rate = 0.1, max_depth = 7, n_estimators = 100, subsample = 1.0 |
| **Gradient Boosting** | ML | learning_rate = 0.05, max_depth = 5, n_estimators = 200, subsample = 0.8 |
| **SVR** | ML | C = 10, gamma = scale, epsilon = 0.05 |
| **LSTM** | DL | Units (Layer 1) = 64, Units (Layer 2) = 32, Dropout = 0.2, Learning Rate = 0.001, Batch Size = 32, Epochs = 20 |
| **GRU** | DL | Units (Layer 1) = 64, Units (Layer 2) = 32, Dropout = 0.2, Learning Rate = 0.001, Batch Size = 32, Epochs = 20 |
| **CNN** | DL | Filters = 64, Kernel Size = 3, Dropout = 0.2, Learning Rate = 0.001, Batch Size = 32, Epochs = 20 |

Table S2 presents the monthly averages of all environmental variables over the 2023-2025 study period. SPEI values reveal a distinct seasonal pattern: negative values (drought) from November to May, with the lowest SPEI (-1.273) occurring in April, and positive values (wet) from June to October, peaking in July (1.498). Temperature follows expected seasonal variation, ranging from 16.39°C in January to 28.96°C in July. Rainfall is concentrated in the monsoon months (June-September), with June receiving the highest average rainfall (13.29 mm/day). PM2.5 shows an inverse relationship with rainfall, peaking in January (88.50 µg/m³) during the dry season and reaching its minimum in July (25.35 µg/m³) during the monsoon.

**Table S2:** Monthly averages of environmental variables (SPEI, temperature, rainfall, wind speed, and PM2.5) for Dinajpur district over the 2023-2025 study period. SPEI values reveal a distinct seasonal pattern: negative values (drought) from November to May, with the lowest SPEI (-1.273) in April, and positive values (wet) from June to October, peaking in July (1.498). PM2.5 shows an inverse relationship with rainfall, peaking at 88.50 µg/m³ in January (dry season) and reaching its minimum of 25.35 µg/m³ in July (monsoon).

| **Month** | **SPEI** | **Temperature (°C)** | **Rainfall (mm)** | **Wind Speed (km/h)** | **PM2.5 (µg/m³)** |
| --- | --- | --- | --- | --- | --- |
| January | -0.126 | 16.39 | 0.01 | 9.91 | 88.50 |
| February | -0.214 | 19.62 | 0.27 | 12.19 | 75.10 |
| March | -0.695 | 23.83 | 0.74 | 15.41 | 56.72 |
| April | -1.273 | 27.75 | 1.54 | 17.20 | 47.97 |
| May | -1.034 | 28.28 | 5.88 | 15.75 | 42.32 |
| June | 0.658 | 28.89 | 13.29 | 15.68 | 39.23 |
| July | 1.498 | 28.96 | 9.41 | 14.76 | 25.35 |
| August | 0.874 | 28.25 | 10.67 | 13.53 | 31.45 |
| September | 0.534 | 28.55 | 9.19 | 11.98 | 32.75 |
| October | 0.775 | 26.21 | 4.52 | 11.32 | 48.60 |
| November | -0.503 | 22.73 | 0.66 | 9.48 | 59.12 |
| December | -0.574 | 18.40 | 0.13 | 9.42 | 69.65 |

Table S3 presents the monthly SPEI forecast for 2027 generated using the Weighted Average hybrid model (GBM + RF + XGBoost) without PM2.5. The forecast predicts a distinct seasonal pattern: dry conditions during the pre-monsoon period (March-April, SPEI: -0.852 to -0.976), followed by wet conditions during the monsoon season (June-September, SPEI: +0.620 to +1.043), and a return to dry conditions in December (SPEI: -0.534). The driest month is predicted to be April (SPEI = -0.976), with a minimum SPEI of -1.547, indicating moderate-to-severe drought conditions. The wettest month is August (SPEI = +1.043), reflecting peak monsoon rainfall. The forecast suggests that approximately 20.5% of days in 2027 may experience drought conditions (SPEI < -0.5), with the majority occurring during March-April and December.

**Table S3:** Monthly SPEI forecast for 2027 with mean, minimum, and maximum values, and drought status classification for each month.

| **Month** | **Mean SPEI** | **Min SPEI** | **Max SPEI** | **Status** |
| --- | --- | --- | --- | --- |
| January | -0.207 | -0.309 | -0.108 | Slightly Dry |
| February | -0.301 | -0.485 | -0.114 | Slightly Dry |
| March | -0.852 | -1.384 | -0.309 | Dry |
| April | -0.976 | -1.547 | -0.184 | Dry |
| May | -0.007 | -1.049 | 0.792 | Slightly Dry |
| June | 0.939 | 0.380 | 1.236 | Wet |
| July | 0.940 | 0.489 | 1.531 | Wet |
| August | 1.043 | 0.497 | 1.381 | Wet |
| September | 0.620 | 0.354 | 1.127 | Wet |
| October | 0.554 | 0.169 | 1.394 | Wet |
| November | 0.286 | 0.131 | 0.597 | Normal |
| December | -0.534 | -0.884 | -0.127 | Dry |

**Note:** SPEI values indicate drought/wet conditions: SPEI < -1.0 (drought), -1.0 to -0.5 (dry), -0.5 to 0 (slightly dry), 0 to 0.5 (normal), 0.5 to 1.0 (wet), > 1.0 (very wet). The forecast was generated using historical climatology (2023-2025) as a baseline scenario with the Weighted Average model (weights: GBM=0.546, RF=0.408, XGB=0.046). PM2.5 was excluded from the model and forecast based on ablation study results.
